# Characterization of gut microbiota associated with clinical parameters in intrahepatic cholestasis of pregnancy

**DOI:** 10.1186/s12876-020-01510-w

**Published:** 2020-11-23

**Authors:** Rong Li, Xuehai Chen, Zhongzhen Liu, Yan Chen, Chuan Liu, Lingfei Ye, Liang Xiao, Zhenjun Yang, Jian He, Wen-Jing Wang, Hongbo Qi

**Affiliations:** 1 Department of Obstetrics, The First Affiliated Hospital of Chongqing Medical University, No. 1 Youyi Road, Yuzhong District, Chongqing, 400016 People’s Republic of China; 2 BGI-Shenzhen, Build 11, Beishan Industrial Zone, Yantian District, Shenzhen, 518000 People’s Republic of China; 3 State Key Laboratory of Maternal and Fetal Medicine of Chongqing Municipality, Chongqing Medical University, Chongqing, 400016 People’s Republic of China; 4 International Collaborative Laboratory of Reproduction and Development of Chinese Ministry of Education, Chongqing Medical University, Chongqing, 400016 People’s Republic of China; 5 China National GeneBank, BGI-Shenzhen, Shenzhen, 518000 People’s Republic of China; 6 BGI-Chongqing Clinical Laboratory, BGI-Shenzhen, Chongqing, 401120 People’s Republic of China

**Keywords:** Intrahepatic cholestasis, Gut microbiota, 16S rRNA, Preterm delivery, Pregnancy

## Abstract

**Background:**

Intrahepatic cholestasis of pregnancy (ICP) is a liver disorder that specifically occurs in pregnancy. Elevated levels of liver transaminases aspartate aminotransferase, alanine aminotransferase and serum bilirubin levels are common biochemical characteristics in ICP. The disorder is associated with an increased risk of premature delivery and stillbirth. The characterization of the potential microbiota in ICP could go a long way in the prevention and treatment of this pregnancy disease.

**Methods:**

A total of 58 patients were recruited for our study: 27 ICP patients and 31 healthy pregnant subjects with no ICP. The V3 and V4 regions of the 16S rDNA collected from fecal samples of both diseased and control groups were amplified. 16S rRNA gene amplicon sequencing was then performed on gut microbiota. Sequencing data were analyzed and the correlation between components of microbiota and patient ICP status was found. Related metabolic pathways, relative abundance and significantly different operational taxonomic units (OTUs) between ICP and controls were also identified.

**Results:**

Elevated levels of total bile acid, ALT, AST, Dbil and Tbil were recorded or observed in ICP subjects as compared to the control. Gut microbiota in pregnant women was dominated by four major phyla and 27 core genera. PCoA analysis results indicated that there was no significant clustering in Bray–Curtis distance matrices. Our results showed that there was a correlation between specific OTUs and measured clinical parameters of pregnant women. Comparison at the different taxonomy levels revealed high levels of abundance of *Blautia* and *Citrobacter* in ICP patients. At the family level, *Enterobacteriaceae* and *Leuconostocaceae* were higher in ICP patients. 638 KEGG Orthologs and 138 pathways significantly differed in the two groups. PLS-DA model with VIP plots indicated a total of eight genera and seven species were key taxa in ICP and control groups.

**Conclusions:**

Our research indicated that although there was no significant clustering by PCoA analysis, patients with ICP have increased rare bacteria at different phylogenetic levels. Our results also illustrated that all 638 KEGG Orthologs and 136 in 138 KEGG pathways were less abundant in ICP patients compared to the controls.

## Background

Intrahepatic cholestasis of pregnancy (ICP) is a common liver disease that occurs during pregnancy. Globally, ICP incidence is reported to occur between 0.2 and 2% depending upon the sample region and ethnicity [1]. Typical symptoms of ICP include itching without a rash that is typically localized to the soles of the feet and palms of the hands. Symptoms also include elevated levels of both liver enzymes and serum bilirubin. Fetal complications are more significant compared to maternally associated complications. In a large prospective national cohort study in the United Kingdom in 2014, women with severe ICP had significantly elevated risks of preterm delivery, stillbirth, and admissions for treatment into neonatal units as compared to control pregnant subjects [2]. Other symptoms that can affect the fetus include meconium-stained amniotic fluids, neonatal depression, and respiratory distress syndrome [3–5].

Although underlying mechanisms of ICP are not fully understood, several factors have been identified as being important. Reproductive hormones that encompass estrogens and progesterones have been implicated in the dynamics of the pathogenesis of ICP. Several studies that have examined data for animal-based models have unveiled the dynamics behind cholestatic effects of estrogen and its impact on hepatotoxicity [6–8]. Researchers also found that the levels of progesterone metabolites were higher in ICP afflicted patients as compared to unafflicted patients, implying an adverse association [9]. Ding et al. reported a higher risk of recurrence of ICP in patients with a family history (92%) as compared to their counterpart sporadic patients (40%) [10]. Furthermore, genetic variants involved in the dynamics of bile acid synthesis and in transport pathways have been implicated in ICP progression. Mutations in the hepatocellular transport protein ABCB4 (MDR3), have been reported in more than 15% of ICP cases [11]. There is also evidence that environmental factors also play important roles in the dynamics of ICP. A cross-sectional cohort study that recruited patients in Chile indicated that the prevalence of ICP was associated with seasonal variation, with the lowest recorded cases in summer months. This seasonal variation was found to coincide with higher levels of plasma Selenium concentrations in summer compared to other months, supporting the implication that nutrition is an important factor in the pathogenesis of ICP [12].

The relationship between gut microbiota and health has been increasingly extensively studied in recent years. As one of the most important factors related to individual health, gut microbiota has been implicated to play important roles in the dynamics of metabolism and immunity of hosts [13, 14]. Some species of gut microbiota have been reported to synthesize vitamins as well as metabolize bile acids and sterols to benefit the hosts [15–18]. Dysbiosis is related to various diseases related to cholestasis, including cirrhosis, cholangitis and etc.[19–21].

Crosstalk between gut microbiota and metabolism of bile acids has been extensively studied in recent times. Gut microbiota are involved in several processes that contribute to the metabolism of bile acids. Ridlon et al. reported that some bacteria mainly from the *Clostridium* and *Eubacterium* genera belonging to the Firmicutes phylum regulate CYP7A1, CYP7B1, and CYP27A1 [22], which play major roles in the metabolism of deconjugated primary bile acids into secondary bile acids through a series of enzymatic reactions. This occurs when there is deconjugation of glycine or taurine from bile acid which subsequently prevents its re-uptake by the small intestines resulting in entry of the aforementioned into the large intestines [23]. Gut microbiota can also regulate bile acid synthesis indirectly via their influences upon receptors, such as FXR and FGF19 [22]. Bile acids in their emulsifying nature are reported to most likely possess the ability to destroy bacterial membranes, thus increasing the transcription of anti-microbial factors through FXR, iNOS and IL-18 to induce an immune response [24]. The dysregulation of microbiota-bile acid interactions also occurs in pathological states, including diet-induced obesity [25], cholestatic liver disease [26], gastrointestinal inflammation, and carcinogenesis [27].

Cirrhosis of the liver which is characterized by severe scarring of the liver and poor liver function is a typical model to illustrate the interrelation of biliary acids—portal blood—gut microbiota axis. In liver cirrhosis, increases in primary bile acid and cholic acid levels cause a dramatic shift toward the *Firmicutes* and lead to increased production of harmful secondary bile acid deoxycholic acid. The *Firmicute* microbiome are reported to cause inflammation, further suppressing the synthesis of bile acids in the liver, leading to a positive-feedback mechanism and progression of the pathology [20, 28].

Fecal samples from 27 pregnant women diagnosed with ICP and 31 healthy women as the control samples were collected in our study. DNA samples from the stool samples of the subjects were extracted which was followed by 16S rRNA sequencing using the amplicons from V3 and V4 region for all qualified DNA samples. Study and examination of potential changes in microbial diversity may provide a better understanding of the progression of ICP and thus lead to better preventive measures and treatment options for patients diagnosed with ICP.

## Methods

### Study participants

This study was performed at the First Affiliated Hospital of Chongqing Medical University, China, between May 2015 and February 2016 with approval of all study aspects and granted from the Ethics Committee of The First Affiliated Hospital of Chongqing Medical University (No. 201530) and all study subjects involved the study. Written informed consent was also obtained from all participating patients.

Samples were collected from 27 patients that had been diagnosed with ICP. ICP diagnosis was done according to the following criteria: severe pruritus without rash; notably elevated concentrations of maternal serum bile acids (> 10 μM); absence of definitive itching-causing diseases; absence of other liver-damaging diseases, such as gallstones, hepatotoxic drug consumption, hepatitis, and inflammatory bowel diseases among others; no smoking or drinking histories and no antibiotics treatment from the onset of pregnancy till the fecal sample collection. Thirty-one age and BMI matched pregnant women unafflicted by ICP were recruited as controls for the study. All women included were Han nationality, without hepatitis B or pregnancy-induced hypertension (PIH), gestational diabetes mellitus (GDM), pre-eclampsia (PE) or other pregnancy-related syndromes.

### Sampling

Stool samples from each of the subjects were collected after ICP diagnoses were confirmed during pregnancy, at consultations and before delivery (for the control samples). Stool samples collected had a normal appearance. After collection, samples were stored at − 80 °C until further processed. Blood samples were also collected after patients had fasted to allow comparative examinations of biochemical parameters including alanine aminotransferase (ALT), aspartate aminotransferase (AST), total serum bilirubin (Tbil), direct bilirubin (Dbil), indirect bilirubin (Ibil) and total bile acid.

### Stool DNA extraction and sequencing

DNA was extracted from stool samples following standard protocols and procedures [29]. We targeted and quantified the expression level of amplicons amplified from the V3 and V4 regions of 16S rDNA for all qualified DNA samples. The primers used to amplify the region are: 341F: ACTCCTACGGGAGGCAGCAG and 806R: GGACTAC(A/T/C)(A/C/G)GGGT(A/T)TCTAAT. The amplicons were sequenced using the Miseq platform and 300-PE-cycles based upon standard protocols described in the literature [29].

### Bioinformatics and statistical analysis

The quality of sequenced reads was assessed with the use of an in-house developed pipeline, which filtered the low-quality data, ambiguous bases, low complexity of reads, and adapter reads as previously described [29]. PE-reads with acceptable levels of quality were then assembled into tags. Operational taxonomic units (OTUs) were clustered using a ≥ 97% similarity threshold for tags with Uparse (version 7.0.1090) using all default settings in the Uparse OTU analysis pipeline program [30]. OTUs were taxonomically annotated using Ribosomal Database Project (RDP, release 11) with a bootstrap cutoff of 80% similar to previous similar studies [31, 32]. Alpha diversity was calculated using Mothur (version 1.31.2) [33]. Corresponding rarefaction curves and box graphs or histograms were plotted by using R statistics software [34]. A particular number of reads were drawn at a time. The initial amount was 1000, followed subsequently by addition of 8,000 reads for each cycle with the highest number of reads at 81,000. The number of iterations per round was 10. The number of OTUs obtained each time was recorded and the corresponding rarefaction curves were plotted.

Beta diversity was measured by Bray–Curtis with the function “beta_diversity.py” in the QIIME pipeline [35]. Principal coordinate analysis (PCoA) analysis was performed using QIIME based on the Bray–Curtis distance. The results from PCoA were plotted using GraphPad Prism 5 software, and the 95% confidence interval ellipse was drawn by ggplot2 [36]. A partial least squares discriminant analysis (PLS-DA) with a variable importance in projection (VIP) plot [37] was performed to determine possible differences in OTUs between ICP patients. This would help in predicting the functional contents of the metagenome. The key genera with VIP > 1.6 were considered important contributors to the model. The KEGG Orthologs and pathway analysis were done by Picrust2.

All biochemical parameters were expressed as the boxplots. Non-parametric Mann–Whitney tests with resultant *p*-values ≤ 0.05 were considered as statistically significant between comparisons of ICP patients and controls. The relative abundance at 95% confidence intervals for differences between ICP patients and controls at a series of taxonomic levels was calculated by using a non-parametric Mann–Whitney test for determination of the false discovery rate (FDR, n = 6). Pearson's correlation coefficients between OTUs and six biochemical parameters (ALT, AST, total bile acid, Tbil, Ibil, and Dbil) were quantified and compared using the cor.test function in the R Statistics suite with all default parameters. The Geom boxplot and geom jitter functions in the ggplot2 package in R statistics were utilized in drawing results for the six biochemical parameters.

## Results

**Fig. 1:**
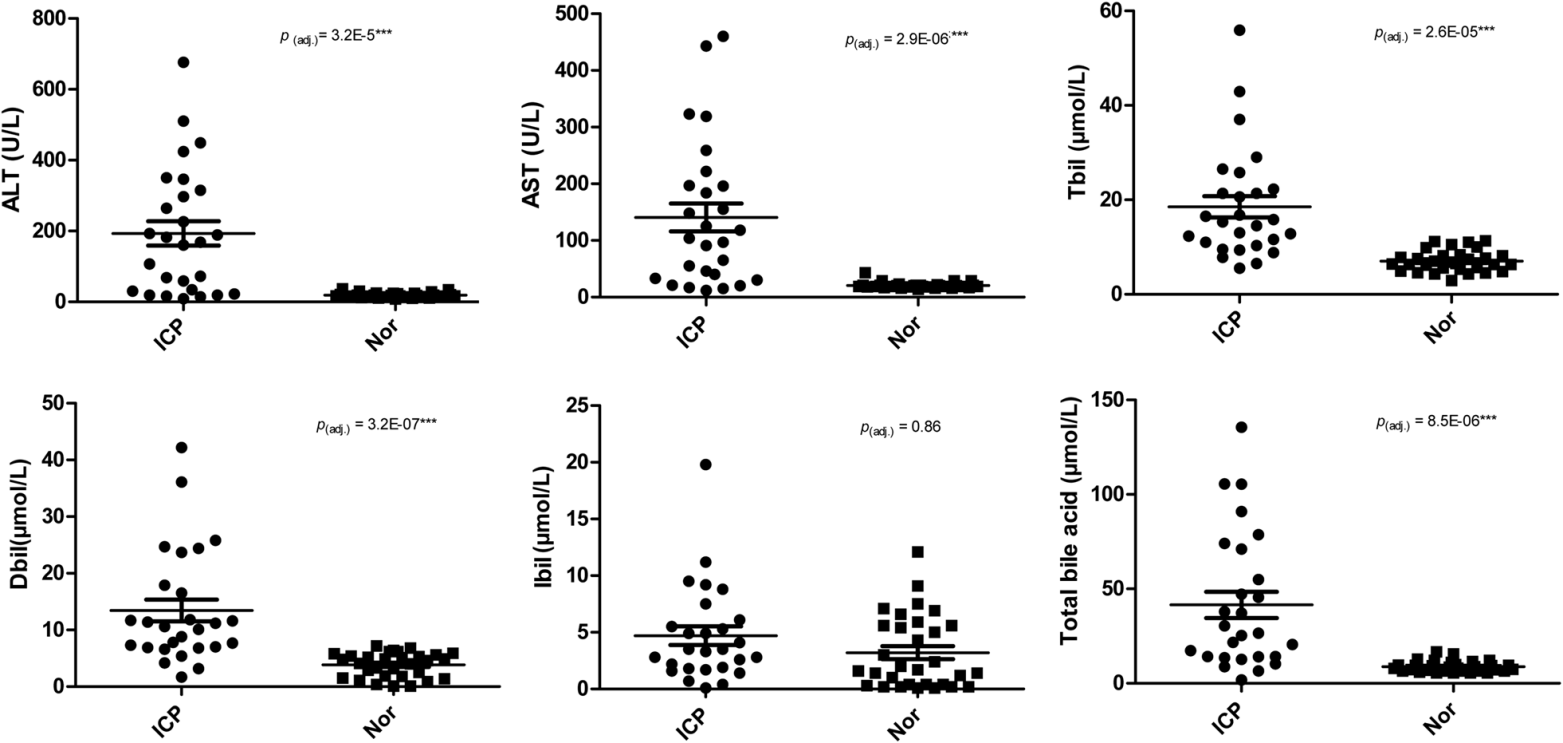
The dot plots of ALT, AST, total bile acid, Dbil, Ibil, and Tbil values in ICP patients and healthy controls, with dots indicating each individual. *AST* aspartate aminotransferase, *ALT* alanine aminotransferase, *Dbil* direct bilirubin, *Ibil* indirect bilirubin; *Tbil* total serum bilirubin. The dots indicate the ICP individuals and squares indicate the control individuals. Bonferroni correction for Mann–Whitney U test is compared between ICP patients and controls

In total, 27 ICP patients and 31 controls were enrolled in our study. Basic clinical information for subjects is summarized in Table 1 and Fig. 1. Age brackets were similar and in very close range for both ICP and control groups. ICP patients and control subjects were sampled at ~ 35.0 and ~ 39.4 weeks respectively. Mean values of total bile acid, ALT, AST, Dbil, and Tbil were all significantly higher in ICP patients than in the control group (Fig. 1, Table 1). Ibil mean values for ICP patients were relatively higher than the value in controls (4.7 ± 0.8 versus 3.2 ± 0.6).

**Table 1:** Study subjects characteristics

	ICP (n = 27)		Control (n = 31)		*p* value (adj.)
	Mean ± SE	IQR	Mean ± SE	IQR	
Age (years)	29.0 ± 1.0	7	29.0 ± 0.79	6	0.5573
Sampling pregnancy weeks (week)	35.0 ± 0.38	3.5	39.4 ± 0.26	2	1.97E−09
Total bile acid (μmol/L)	41.6 ± 6.9	57.1	8.8 ± 0.5	3.3	8.51E−06
Tbil (μmol/L)	18.5 ± 2.3	12	7.0 ± 0.4	2.9	2.56E−05
Ibil (μmol/L)	4.7 ± 0.8	4.3	3.2 ± 0.6	5.2	0.8637796
Dbil (μmol/L)	13.4 ± 1.9	11	3.9 ± 0.4	3.8	3.16E−07
AST (U/L)	140 ± 24.6	164	20.7 ± 1.0	3	2.90E−06
ALT(U/L)	193.3 ± 34.4	285	18.6 ± 1.2	9	3.22E−05

The *p* values were calculated using Mann–Whitney U test with a Bonferroni correction

*Tbil* total serum bilirubin, *Ibil* indirect bilirubin, *Dbil* direct bilirubin, *AST* aspartate aminotransferase, *ALT* alanine aminotransferase, *SE* standard error

### Characterizing gut microbiota in women with ICP and controls

16S rRNA amplicon-based microbiome analysis was performed on stool samples from 27 ICP patients and 31 healthy controls. After sequencing using the Miseq platform and preliminary data processing, clean reads were assembled into tags. Similar numbers of tags (on average 89,265 ± 17,127 tags for each ICP patient and 90,964 ± 13,947 tags for each control) were clustered into OTUs. A total of 875 OTUs were found from all samples. There was no significant difference in the identified OTUs between the two groups (median = 234 for ICP patients and 238 for controls, *p* = 0.3944, Additional file 1: Figure S1A). Alpha diversity analyses revealed that the number of OTUs in ICP patients was 9.8% lower than controls (*p* = 0.0165). Alpha rarefaction curves of numbers of observed OTUs for both patient groups showed a gradual leveling off by 60,000 sequences. The average number of reads for each sample was 287,000, which was much more than 60,000 (Additional file 1: Figure S1B).

Collectively, the composition of gut microbiota of pregnant women was dominated by four major phyla: *Firmicutes*, *Bacteroidetes*, *Actinobacteria*, and *Proteobacteria*, all of which existed in more than 95% of these samples. At the genus level, 27 core genera that included *Faecalibacterium, Streptococcus* and *Escherichia* existed in more than 95% of the samples from pregnant women (Additional file 1: Table S1).

**Fig. 2:**
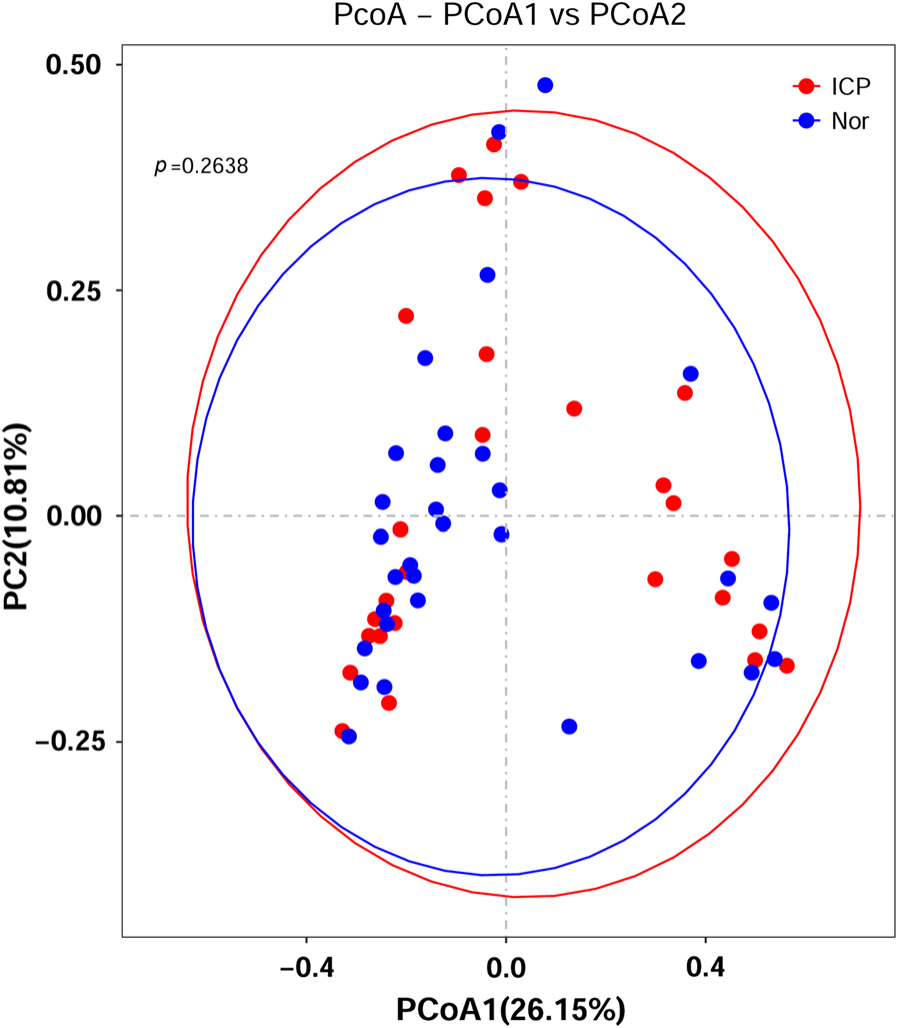
PCoA analysis plots of Bray–Curtis distances between ICP patients and controls. Dots in red indicate ICP patients and dots in blue indicate controls. The *p *value was tested with ANOSIM (*p* = 0.26). Ellipses are drawn at 95% confidence intervals for the ICP and control groups

To assess the structural similarities in the gut microbiota communities between the ICP patients and controls, a principle coordinates analysis (PCoA) was generated based on Bray–Curtis distance. The analysis is primarily based on the number of OTUs and not the specific taxonomic annotation. The PCoA results showed no separation of the ICP patients from the control group, indicating that the main composition of the gut microbiome of the ICP group was not significantly different from the control group (Fig. 2, ANOSIM, *p* = 0.26).

The alpha-diversity between ICP patients and controls was also assessed. The five measures (Chao, ace, sobs, Shannon, Simpson) were used to analyze the abundance and diversity of microbiota within samples. None of the five measures showed any significant differences between the two groups (Additional file 1: Figure S2 and Table S2).

**Fig. 3:**
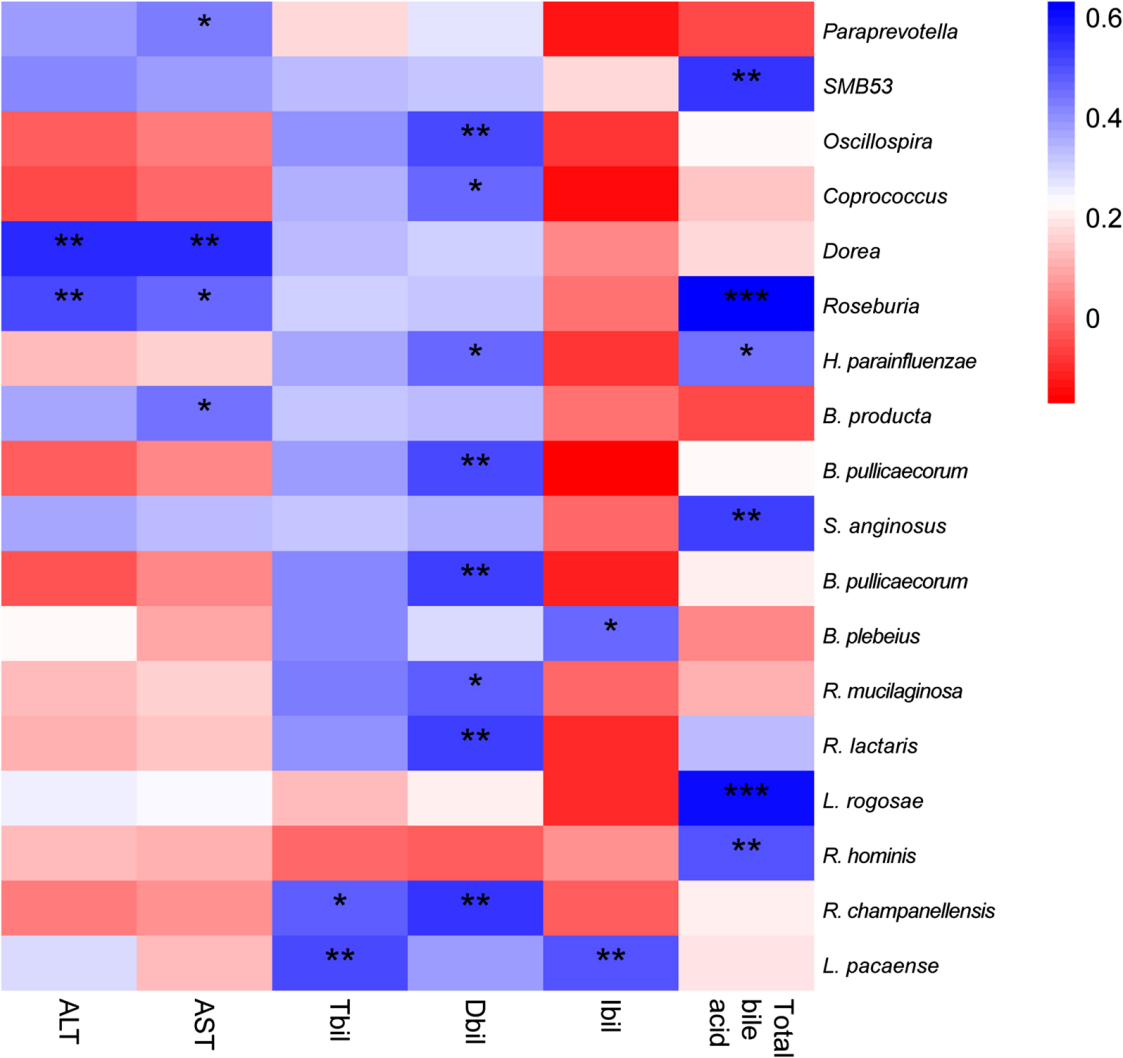
Significant correlations between OTUs and clinical parameters on both ICP patients and controls. Pearson's correlation coefficients were tested between OTUs and six biochemical parameters. The heat map shows the positive-to-negative correlations

The correlation coefficient between OTUs and clinical parameters was examined. The correlation between bacterial species (with prevalence ≥ 20%) and clinical parameters in ICP group was also assessed and no bacteria was identified or found. A subsequent slight change in conditions was made to aid observation of the correlated OTUs for clinical parameters in both ICP patients and controls. We observed the associations between specific microbiota and some liver parameters. Results showed that:

1. *Roseburia* and *Dorea* were positively associated with ALT.
2. *SMB53*, *Roseburia*, *H. parainfluenzae*, *S. anginosus*, *L. rogosae* and *R. hominis* showed some encouraging association with total bile acid.
3. *O. coprococcus*, *H. parainfluenzae*, *B. pullicaecorum*, *R. mucilaginosa*, *R. lactaris*, *R. champanellensis* were associated with Dbil.
4. Associations between *Paraprevotella*, *Dorea*, *Roseburia* and *B. product* with AST, *R. champanellensis* and *L. pacaense* with Tbil and *B. plebeius* and *L. pacaense* with Ibil were observed as shown in Fig. 3.

**Fig. 4:**
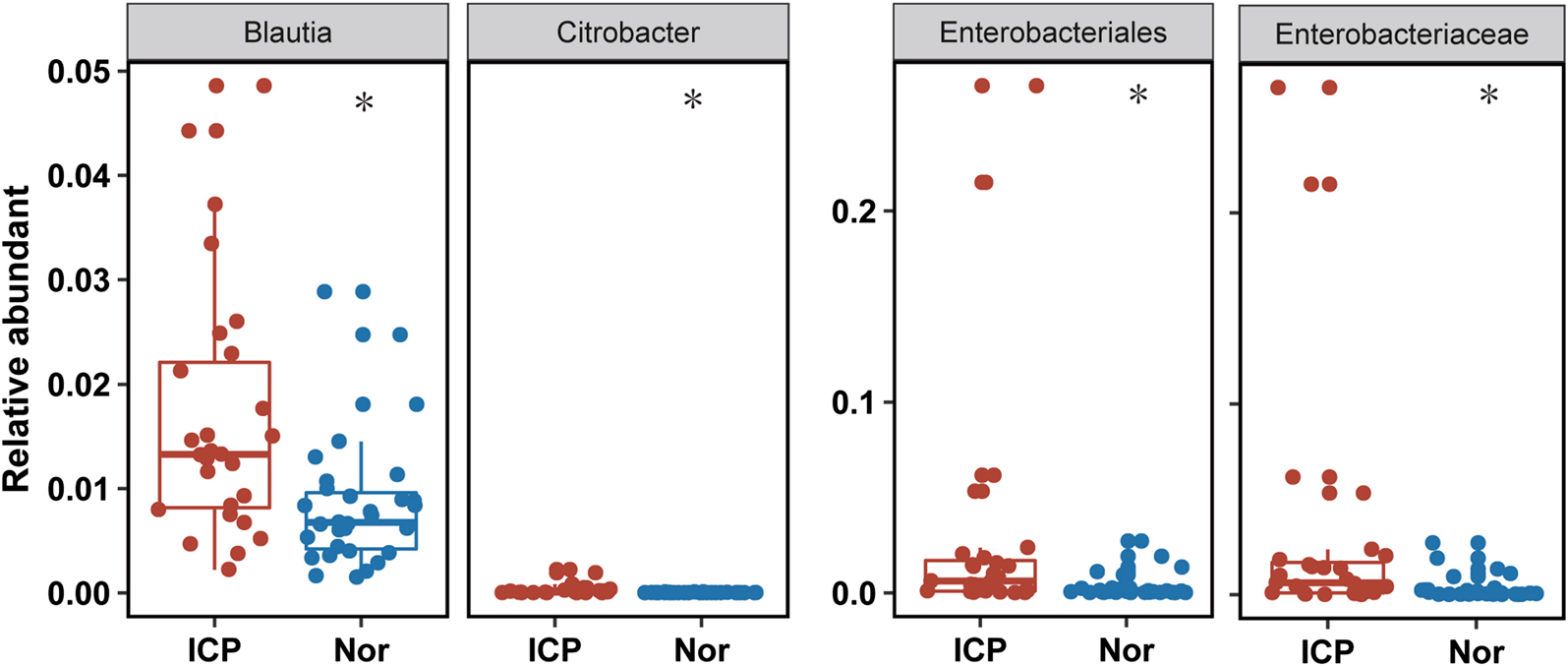
Differential relative abundance between ICP patients and healthy controls on representative taxa. Boxes/dots in red are for ICP patients and in blue for control groups. The *p* value is calculated by Mann–Whitney U test with Bonferroni correction

The Mann–Whitney U test (with an FDR correction) was then utilized in finding the differential relative abundances of bacteria at different taxonomy levels. As shown in Table 2, the abundance of *Blautia*, *Citrobacter* and *Streptococcus* at the genus level, was significantly higher in ICP patients than in controls. Similarly at the family level, *Enterobacteriaceae*, *Leuconostocaceae* and *Streptococcaceae* were higher in ICP patients. *Bacilli* and *Gammaproteobacteria* and *Enterobacterials* and *Lactobacillales* levels were higher at the class and order levels respectively. *Streptococcus luteciae,* at the species level was higher in ICP patients (Fig. 4, Table 2). All these bacteria were considered to be rare (mean relative abundance < 5%) in both the ICP patients and controls.

**Table 2:** Differential relative abundances of bacteria in six levels

Items	Mean (ICP)	SD (ICP)	Mean (Nor)	SD (Nor)	*p *value	FDR	
*Bacilli*	0.0038	0.0058	0.0014	0.0018	0.0055	0.015548	Class
*Gammaproteobacteria*	0.0311	0.0636	0.0052	0.0085	0.0070	0.022667	
*Enterobacteriales*	0.0282	0.0633	0.0033	0.0063	0.0021	0.032923	Order
*Lactobacillales*	0.0033	0.0057	0.0011	0.0017	0.0007	0.008636	
*Enterobacteriaceae*	0.0282	0.0633	0.0033	0.0063	0.0021	0.0282	Family
*Leuconostocaceae*	8.013E−05	2.095E−04	3.314E−06	1.402E−05	9.065E−04	0.006011	
*Streptococcaceae*	0.0029	0.0055	0.0008	0.0009	0.0041	0.006011	
*Blautia*	0.0168	0.0121	0.0084	0.0062	0.0009	0.026867	Genus
*Citrobacter*	3.087E−04	5.472E−04	8.751E−06	1.978E−05	2.218E−05	0.007293	
*Streptococcus*	2.826E−03	5.532E−03	7.179E−04	8.808E−04	1.782E−02	0.026867	
*Streptococcus luteciae*	2.148E−04	3.561E−04	7.496E−05	1.036E−04	1.057E−02	2.148E−04	Species

The *p* value is calculated by using Mann–Whitney U test with an FDR (n = 6)

*SD* standard deviation

**Fig. 5:**
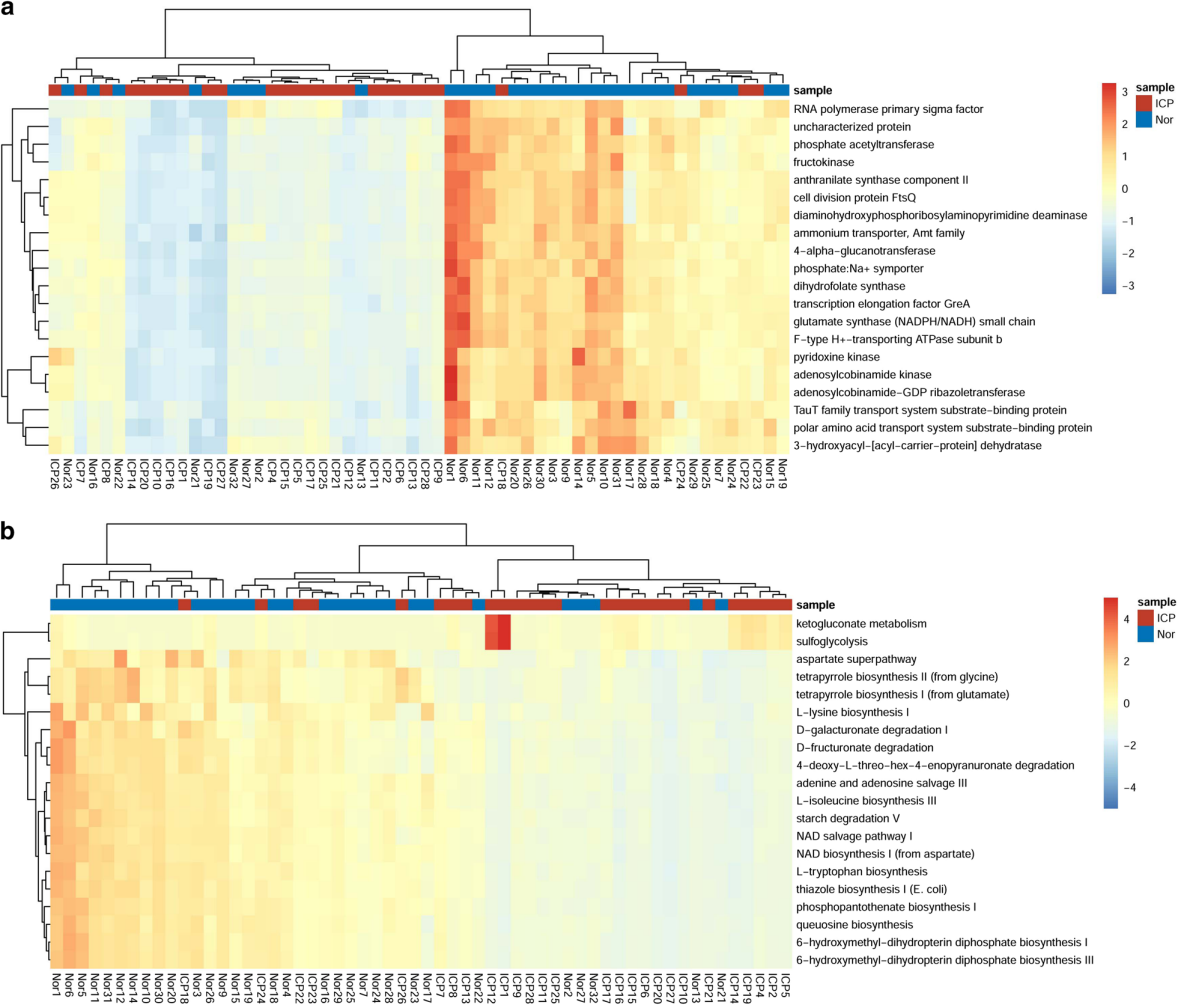
The top 20 differential KEGG orthologs and pathways. **a** The heatmap of the top 20 differential KEGG orthologs clustered by relative counters of OTUs. **b** The heatmap of the top 20 differential KEGG pathways clustered by relative counters of OTUs. The KEGG orthologs and pathways are analyzed by RHeartmap in Picrust2, the *p* values are calculated by Mann–Whitney U test with Benjamini–Hochberg procedure

The functional profile of the gut microbiota from ICP patients and controls was also explored. The total OTUs were normalized by 16S rDNA copy number. The KEGG orthologs, Enzyme Classification and metagenomic functions were predicted from the KEGG pathways. A total of 638 KEGG Orthologs were identified significantly different between ICP patients and controls, all of which were less abundant in ICP patients (*P* < 0.05; Additional file 2). A total of 138 pathways were identified significantly different between the two groups, whereas 136 pathways were significantly more abundant in controls (*P* < 0.05; Additional file 3). Of these KEGG Orthologs, the greatest difference was observed among RNA polymerase primary sigma factor between the two groups (Fig. 5a). To the KEGG pathways, the greatest difference was observed among ketogluconate metabolism which was more represented in ICP patients than in controls (Fig. 5b).

**Fig. 6:**
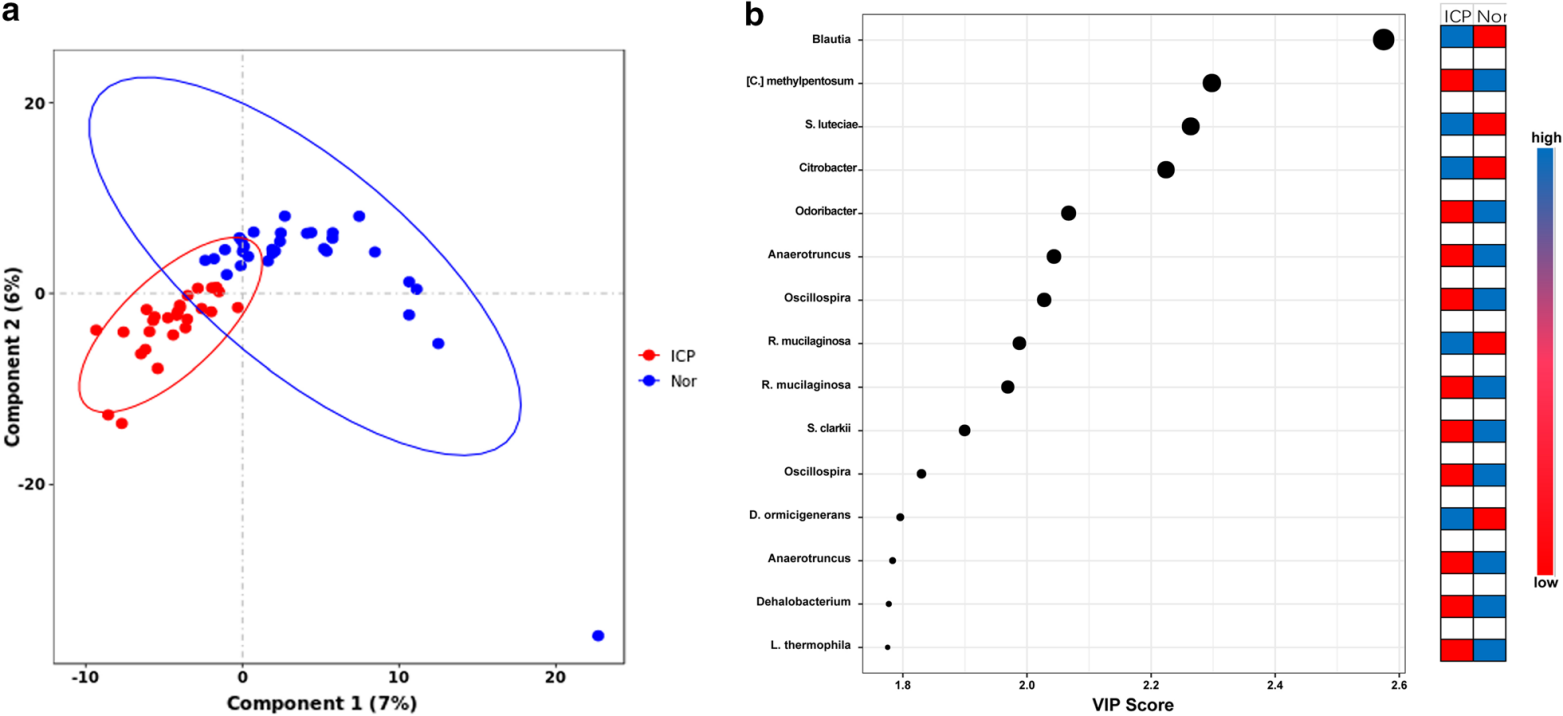
Assessment of partial least squares discriminant analysis (PLS-DA) with variable importance of projection (VIP) scores for relative bacterial abundance in ICP and control groups. **a** The PLS-DA score plots showing model discrimination between ICP and control groups. **b** The VIP plot indicating the most discriminating bacteria in the descending order of importance. PLS-DA and VIP scores were assessed using MetaboAnalyst 3.0. Ellipses represent 95% confidence intervals for each group on PLS-DA plots. VIP scores reflect the degree of importance of a bacterium, with values > 1.6 seen as driving the calculated discrimination

The contribution of the taxa to the discrimination between ICP patients and controls was assessed by PLS-DA model and VIP score. PLS-DA score plot showed model discrimination between ICP patients and controls. A total of eight genera and seven species were recorded with VIP > 1.6. These were identified as key genera/species in ICP and control group. Of these, five were more abundant in ICP patients whereas the remaining 10 were more abundant in controls. *Blautia* genus (~ 2.6) and *[C.] methylpentosum* species (~ 2.3) recorded the highest VIP scores (Fig. 6).

## Discussion

In this study, we analyzed gut microbiota in the third-trimester of sections of ICP pregnant women and healthy control pregnant women. As at the time of our study, the relationship between gut microbiota and ICP was studied for the first time and no previous reports had been made to our knowledge. Correlation coefficients between OTUs and clinical parameters (liver) of ICP patients and controls at different taxonomic levels were determined. In addition, we assessed the contribution of the genera or species to the discrimination between ICP patients and controls. It was observed when a collective study of bacteria from both study groups done that, *Firmicutes*, *Bacteroidetes*, *Actinobacteria,* and *Proteobacteria* were the dominant phyla, whereas 27 core genera including *Faecalibacterium, Streptococcus, Escherichia* were the dominant genera in both groups. The compositions of these taxa were found to be in accordance with a previous study by Koren et al., which focused on changes of microbiota during pregnancy. In their study, the relative abundances of *Proteobacteria* and *Actinobacteria* increased as the pregnancy progressed. In our study, OTUs including members of the *Enterobacteriaceae* family and *Streptococcus* genus were dominant in the third trimester which was similar to previous reports by Koren et al. [38]. In a cohort study of 314 young Chinese individuals conducted by Zhang et al., a list of 16 abundant genera were reported in their fecal samples. Eleven of these were included in our core genera list, further validating our data [39].

While few studies focused upon the flora making up the gut microbiota in patients with ICP, some teams have studied the metagenomes in bile acid-related abnormity or liver diseases. A series of studies examined gut microbiota for patients with cirrhosis [17, 20, 28, 40]. It was reported that when levels of observed bile acid entering the intestine that were low, levels of *Enterobacteriaceae* (the only one family belonging to *Enterobacteriales*) was found to have increased. In a study that examined primary sclerosing cholangitis (PSC), it was observed that there were high levels *Blautia* when there was inhibition of bile released in the small intestines [41]. We obtained similar results in our experiments. Intestinal bile acid is one of the major regulators of gut microbiota and inhibition of the entrance of bile acid to intestines causes bacterial dysbiosis, as gram-positive members such as *Rumminococcaceae* and members of *Clostridium cluster* XVIa, which are involved in secondary fecal bile acid production and anti-inflammatory response, were inhibited [23]. Contrastingly, pro-inflammatory and potentially pathogenic taxa, including *Enterobacteriaceae*, increased [40].

Bile acids affect gut microbiota composition directly through antimicrobial effects or indirectly through impacts upon FXR-dependent antimicrobial peptides. As one of the components of the pool of bile acids, deoxycholic acid (DCA), has a strong effect upon inhibiting the growth of the microbiome and acts as a detergent upon bacterial membranes [42].

In our study, women with ICP were sampled at a median time of pregnancy of 35.0 weeks (i.e. before full-term) and control women were sampled at a median of 39.4 weeks (i.e. at term). A previous study that examined temporal variation in the composition of human microbiota during pregnancy evaluated the communities sampled in consecutive weeks through delivery and found that there were no significant trends over gestational time (*P* > 0.05, *t* test) [43]. Thus, in our study, we felt it was appropriate and reasonable to have collected samples at different time points in the third trimester.

In conclusion, our study presented the first view of research which examined the gut microbiota of ICP afflicted patients. Although the mechanisms and dynamics with regards to how phylogenetic diversity changes gut microbiota in patients afflicted with ICP remain obscure, our findings might provide new diagnostic and treatment strategies during pregnancy for this disease and the associated symptoms. Further studies are needed to identify factors impacting gut bacterial composition in ICP patients to prevent the occurrence and progression of these complications in the third trimester of pregnancy.

## Conclusions

In this study, the fecal microbiota from 27 ICP patients and 31 comparable controls were analyzed by 16S rRNA gene amplicon sequencing. The differential relative abundances of bacteria at different taxonomy levels between ICP patients and controls were compared and the correlation between OTUs and clinical liver characters was explored. The functional profile of the gut microbiota from ICP patients and controls were compared. The contribution of the genera or species to the discrimination between ICP patients and controls was assessed. Our results indicated for the first time that patients with ICP have an altered phylogenetic gut microbiota profile compared with the control group.

## Supplementary information

**Additional file 1**:** Figure S1**. Boxplot and rarefaction curves of operational taxonomic units (OTUs). **a** Boxplot of OTUs of ICP patients and controls. The *p* values are calculated by student’s test. **b** The rarefaction curves of OTUs. The x-axis shows the number of valid sequences per sample and the y-axis shows the observed species (OTUs). The curve in red color represents ICP patients and the curve in green represents controls. **Figure S2**. Box-plots illustrating alpha diversity indices (Chao, ace, sobs, Shannon, simpson) in bacterial microbiota of ICP patients and controls. Median values and interquartile ranges have been indicated in the plots. **Table S1**. The common core phylum-genus in ICP patients and controls. **Table S2**. Alpha diversity index value. The mean and standard deviations of alpha diversity indices (Chao, ace, sobs, Shannon, simpson).

**Additional file 2**. The total list of 638 KEGG Orthologs significantly different between ICP patients and controls.

**Additional file 3**. The total list of 138 KEGG pathways significantly different between ICP patients and controls.

## Data Availability

The datasets generated and analysed during the current study are available in the CNSA (https://db.cngb.org/cnsa/) of CNGBdb repository, with the accession code CNP0000403.

## Abbreviations

ICP: Intrahepatic cholestasis of pregnancy
ALT: Alanine aminotransferase
AST: Aspartate aminotransferase
Tbil: Total serum bilirubin
Dbil: Direct bilirubin
Ibil: Indirect bilirubin
OTUs: Operational taxonomic units
RDP: Ribosomal Database Project
PCoA: Principal coordinate analysis
FDR: False discovery rate
IQR: Interquartile range
PLS-DA: Partial least squares discriminant analysis
VIP: Variable importance in projection
SCFA: Short-chain fatty acids
DCA: Deoxycholic acid
