# Supplementary material for: Characterization of gut microbiota associated with clinical parameters in intrahepatic cholestasis of pregnancy
Source: BMC Gastroenterol. 2020 Nov 23;20:395. doi: 10.1186/s12876-020-01510-w (PMC7682054; doi:10.1186/s12876-020-01510-w)
Supplement: Supplementary file 1 — Additional file 1: Figure S1. Boxplot and rarefaction curves of operational taxonomic units (OTUs). a Boxplot of OTUs of ICP patients and controls. The p values are calculated by student’s test. b The rarefaction curves of OTUs. The x-axis shows the number of valid sequences per sample and the y-axis shows the observed species (OTUs). The curve in red color represents ICP patients and the curve in green represents controls. Figure S2. Box-plots illustrating alpha diversity indices (Chao, ace, sobs, Shannon, simpson) in bacterial microbiota of ICP patients and controls. Median values and interquartile ranges have been indicated in the plots. Table S1. The common core phylum-genus in ICP patients and controls. Table S2. Alpha diversity index value. The mean and standard deviations of alpha diversity indices (Chao, ace, sobs, Shannon, simpson). [file 12876_2020_1510_MOESM1_ESM.pdf]

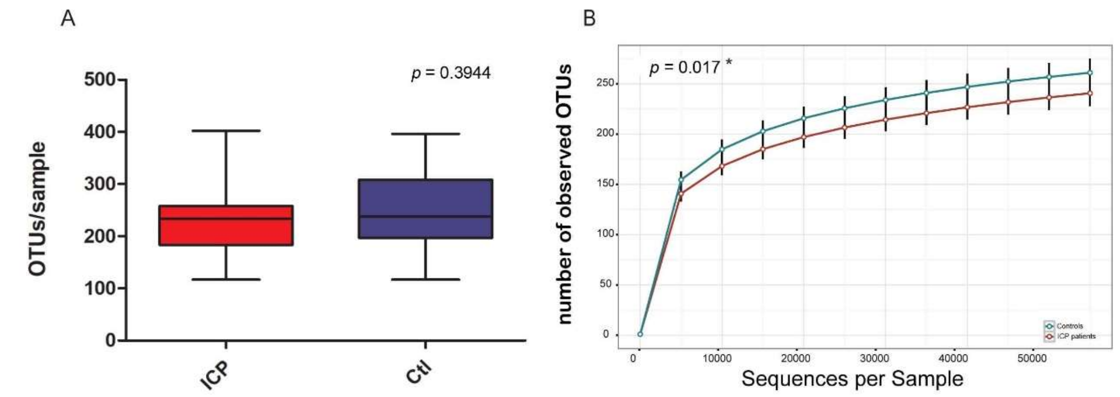

Figure S1. Boxplot and rarefaction curves of operational taxonomic units (OTUs). (A) Boxplot of OTUs of ICP patients and controls. The *p*-values are calculated by student's test. (B) The rarefaction curves of OTUs. The x-axis shows the number of valid sequences per sample and the y-axis shows the observed species (OTUs). The curve in red color represents ICP patients and the curve in green represents controls.

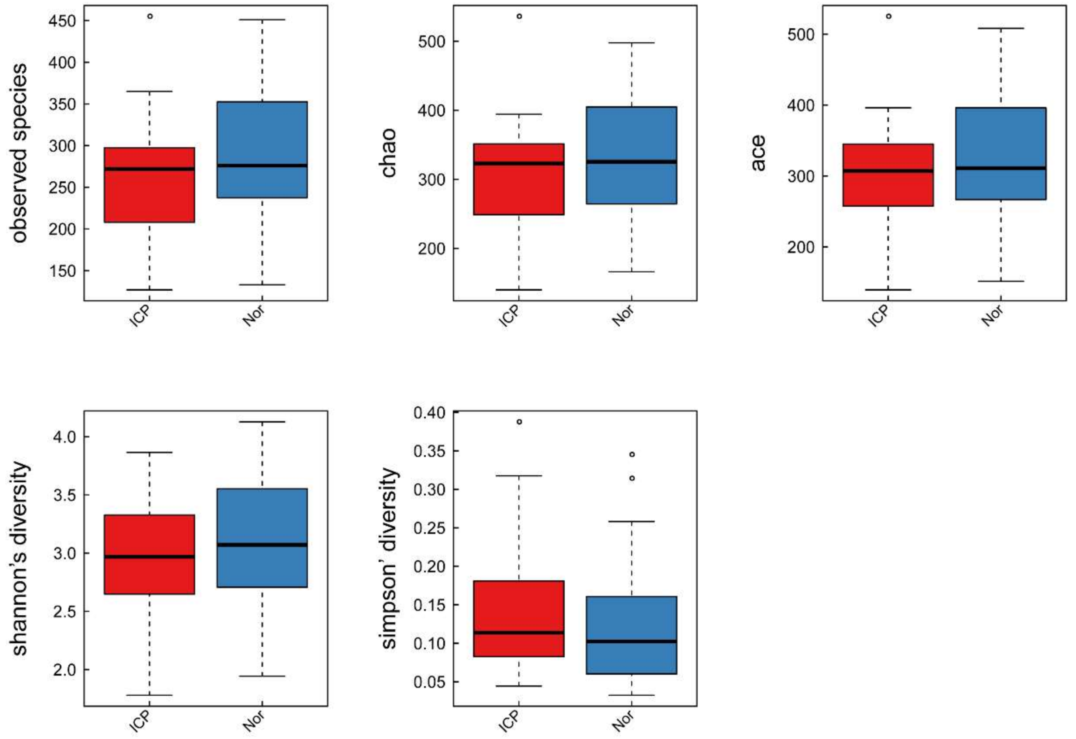

Figure S2. Box-plots illustrating alpha diversity indices (Chao, ace, sobs, Shannon, simpson) in bacterial microbiota of ICP patients and controls. Median values and interquartile ranges have been indicated in the plots.

Table S1. The common core phylum-genus in ICP patients and controls.

the common core phyla in both ICP and control groups
k__Bacteria-p__Actinobacteria
k__Bacteria-p__Bacteroidetes
k__Bacteria-p__Firmicutes
k__Bacteria-p__Proteobacteria
**the common core genus in both ICP and control groups**
k__Bacteria-p__Actinobacteria-c__Actinobacteria-o__Bifidobacteriales-f__Bifidobacteriaceae-g__Bifidobacterium
k__Bacteria-p__Bacteroidetes-c__Bacteroidia-o__Bacteroidales-f__Bacteroidaceae-g__Bacteroides
k__Bacteria-p__Bacteroidetes-c__Bacteroidia-o__Bacteroidales-f__Porphyromonadaceae-g__Parabacteroides
k__Bacteria-p__Firmicutes-c__Bacilli-o__Bacillales-f__Bacillaceae-g__Bacillus
k__Bacteria-p__Firmicutes-c__Bacilli-o__Lactobacillales-f__Streptococcaceae-g__Lactococcus
k__Bacteria-p__Firmicutes-c__Bacilli-o__Lactobacillales-f__Streptococcaceae-g__Streptococcus
k__Bacteria-p__Firmicutes-c__Clostridia-o__Clostridiales-f__-g__
k__Bacteria-p__Firmicutes-c__Clostridia-o__Clostridiales-f__Clostridiaceae-g__Clostridium
k__Bacteria-p__Firmicutes-c__Clostridia-o__Clostridiales-f__Clostridiaceae-g__SMB53
k__Bacteria-p__Firmicutes-c__Clostridia-o__Clostridiales-f__Lachnospiraceae-Other
k__Bacteria-p__Firmicutes-c__Clostridia-o__Clostridiales-f__Lachnospiraceae-g__
k__Bacteria-p__Firmicutes-c__Clostridia-o__Clostridiales-f__Lachnospiraceae-g__Blautia
k__Bacteria-p__Firmicutes-c__Clostridia-o__Clostridiales-f__Lachnospiraceae-g__Coprococcus
k__Bacteria-p__Firmicutes-c__Clostridia-o__Clostridiales-f__Lachnospiraceae-g__Dorea
k__Bacteria-p__Firmicutes-c__Clostridia-o__Clostridiales-f__Lachnospiraceae-g__Lachnospira
k__Bacteria-p__Firmicutes-c__Clostridia-o__Clostridiales-f__Lachnospiraceae-g__Roseburia
k__Bacteria-p__Firmicutes-c__Clostridia-o__Clostridiales-f__Lachnospiraceae-g__[Ruminococcus]
k__Bacteria-p__Firmicutes-c__Clostridia-o__Clostridiales-f__Ruminococcaceae-g__
k__Bacteria-p__Firmicutes-c__Clostridia-o__Clostridiales-f__Ruminococcaceae-g__Butyricicoccus
k__Bacteria-p__Firmicutes-c__Clostridia-o__Clostridiales-f__Ruminococcaceae-g__Faecalibacterium
k__Bacteria-p__Firmicutes-c__Clostridia-o__Clostridiales-f__Ruminococcaceae-g__Oscillospira
k__Bacteria-p__Firmicutes-c__Clostridia-o__Clostridiales-f__Ruminococcaceae-g__Ruminococcus
k__Bacteria-p__Firmicutes-c__Clostridia-o__Clostridiales-f__Veillonellaceae-g__Phascolarctobacterium
k__Bacteria-p__Firmicutes-c__Erysipelotrichi-o__Erysipelotrichales-f__Erysipelotrichaceae-g__
k__Bacteria-p__Proteobacteria-c__Betaproteobacteria-o__Burkholderiales-f__Alcaligenaceae-g__Sutterella
k__Bacteria-p__Proteobacteria-c__Deltaproteobacteria-o__Desulfovibrionales-f__Desulfovibrionaceae-g__Bilophila
k__Bacteria-p__Proteobacteria-c__Gammaproteobacteria-o__Enterobacteriales-f__Enterobacteriaceae-g__Escherichia

Table S2. alpha diversity index value.

#Alpha	mean(ICP)	SD(ICP)	mean(Nor)	SD(Nor)	*p*-value
sobs	264.7778	71.1825	286.129	82.96776	0.38265
chao	309.2285	80.28008	327.8503	90.37645	0.44788
ace	307.6283	76.81137	322.2008	89.97471	0.50554
shannon	2.9594	0.54626	3.1374	0.57948	0.37705
simpson	0.14116	0.08397	0.12164	0.07887	0.2573

The mean and standard deviations of alpha diversity indices (Chao, ace, sobs, Shannon, simpson).
